# Mid-gestation cytokine profiles in mothers of children affected by autism spectrum disorder: a case–control study

**DOI:** 10.1038/s41598-021-01662-z

**Published:** 2021-11-16

**Authors:** Michael Carter, Sophie Casey, Gerard W. O’Keeffe, Louise Gibson, Deirdre M. Murray

**Affiliations:** 1 The Irish Centre for Maternal and Child Health Research, University College Cork, Cork, Ireland; 2 National Children’s Research Centre, Crumlin, Dublin 12, Ireland; 3 Department of Anatomy and Neuroscience, University College Cork (UCC), Cork, Ireland; 4 Department of Paediatrics and Child Health, University College Cork (UCC), Cork, Ireland

**Keywords:** Biological techniques, Immunological techniques, Biomarkers, Cytokines

## Abstract

Autism Spectrum disorder is one of the commonest and most important neurodevelopmental conditions affecting children today. With an increasing prevalence and an unclear aetiology, it is imperative we find early markers of autism, which may facilitate early identification and intervention. Alterations of gestational cytokine profiles have been reported in mothers of autistic children. Increasing evidence suggests that the intrauterine environment is an important determinant of autism risk. This study aims to examine the mid-gestational serum cytokine profiles of the mothers of autistic children from a well-characterised birth cohort. A nested sub-cohort within a large mother–child birth cohort were identified based on a confirmed multi-disciplinary diagnosis of autism before the age 10 years and neuro-typical matched controls in a 2:1 ratio. IFN-γ, IL-1β, IL-4, IL-6, IL-8, IL-17A, GMCSF and TNFα were measured in archived maternal 20-week serum using MesoScale Diagnostics multiplex technology and validation of our IL-17A measurements was performed using an ultrasensitive assay. From a cohort of 2137 children, 25 had confirmed autism before 10 years and stored maternal serum from mid-gestation. We examined the sera of these 25 cases and 50 matched controls. The sex ratio was 4:1 males to females in each group, and the mean age at diagnosis was 5.09 years (SD 2.13). We found that concentrations of IL-4 were significantly altered between groups. The other analytes did not differ significantly using either multiplex or ultra-sensitive assays. In our well-characterised prospective cohort of autistic children, we confirmed mid-gestational alterations in maternal IL-4 concentrations in autism affected pregnancies versus matched controls. These findings add to promising evidence from animal models and retrospective screening programmes and adds to the knowledge in this field.

Autism spectrum disorder (ASD) is an intricate continuum of neurodevelopmental disorders, all of which have an onset in early childhood and persist throughout life. These disorders are characterised by core impairments in social communication, and the presence of restricted and repetitive interests and behaviours^1–4^. There exists within this spectrum a broad range of heterogeneity. Clinical phenotypes vary widely, aetiology remains unclear, and many different comorbidities afflict those with ASD. There is clearly a strong genetic component in many cases with heritability estimates of 50–90%^5,6^, while the apparent male preponderance with rates exceeding that of females three to fourfold, also hints at a strong genetic foundation^7,8^. Yet, even using newer techniques such as ASD-optimised ultrahigh resolution chromosomal microarray, we only find a single gene determinant in approximately 25% of cases^9,10^. A recent study of monozygotic twins (MZ) (who share 100% similar copies of their genetic material) quoted ASD concurrence rates as low as 59% between MZ siblings^11^. Some authors have found that specific HLA-DR (Human leukocyte antigen) subtypes are overexpressed in children with ASD versus controls^12,13^. Despite these advances, we are yet to discover a single gene determinant that can account for more than a small percent of ASD cases. All this suggests that we cannot explain many cases of ASD by genetic factors alone, or at least we cannot explain them using our current understanding of ASD genetics or our current techniques of genetic analysis.

This imperfect picture of ASD genetics has led some to investigate the role of environmental exposures in the aetiology of ASD. Researchers have identified many environmental risks in ASD. Advanced parental age, foetal environmental exposures, perinatal and obstetric events, maternal medication use, smoking and alcohol use, psychosocial hardship, nutrition and toxic exposures have all been implicated as risks in the pathogenesis of ASD^11,14^. Some authors attribute up to 17% of ASD risk to these exposures, yet the exact balance between genetic and environmental determinants and their roles in aetiology remains disputed^11,15^. The current ASD literature suggests that mutations occurring in genes involved in synapse formation, cell adhesion molecule production (such as Cadherins), scaffolding proteins (SHANK proteins), ion channels (sodium, calcium, and potassium channels), and signaling molecules can disrupt regulatory or coding regions and affect synapse formation, plasticity and synaptic transmission^16^.

Cell signaling pathways such as PI3K, PTEN and mTOR interact with synapse influencing targets at multiple levels, mutations affecting these pathways lead to aberrant synaptic protein synthesis and have been shown to influence the development of monogenic (syndromic ASD) as well as non-syndromic ASD^17,18^.

Multiple mechanisms have been proposed through which each of these exposures may exert an influence on genetic and epigenetic risk in ASD, but there are only a handful that are likely to effect abnormal neurodevelopment. Animal models of inflammation and maternal immune activation are particularly well characterised, and have successfully modelled ASD type behaviours and social difficulties in mice, rats and non-human primates^19–21^.

Maternal immune activation (MIA) is defined as an increase in measured levels of inflammatory markers in mothers during pregnancy, and more specifically refers to a triggering of the maternal immune system by infectious or infectious‐like stimuli resulting in an increase in measurable inflammatory markers^22,23^. Through this activation, a cytokine cascade transmits to the foetus, resulting in adverse neurodevelopmental phenotypes and even remodelling or malformations of the developing foetal brain. There have been many studies, which have profiled cytokine, chemokine, immune cell and inflammatory signatures in ASD affected individuals^24–29^. A much smaller number of studies have characterised cytokine profiles in expectant mothers who progressed to give birth to children who develop ASD^30,31^. The few previous studies, which have examined gestational serum, have indicated mid-gestational upregulation in specific pro-inflammatory cytokines or indeed down-regulation in anti-inflammatory cytokines. These findings arise from retrospective examination of stored serum samples from the wide 15–19 week gestational window. None of these studies confirmed a formal psychiatric or multi-disciplinary team diagnosis of ASD, nor did they account for important and relevant underlying maternal inflammatory conditions such as inflammatory bowel disease^32^ and rheumatoid arthritis^33^. Our aim in this study was to measure candidate cytokines at a single specific mid-gestational time-point (20-weeks’ gestation) in a carefully characterised prospectively recruited birth cohort.

## Methods

### Study population

Mother and child dyads were recruited from the Cork BASELINE Birth Cohort Study (Babies after SCOPE: Evaluating the Longitudinal Impact on Neurological and Nutritional Endpoints) (www.baselinestudy.net). In total, recruitment ran for just over three years, from August 2008 to October 2011. The SCOPE Ireland pregnancy cohort (www.scopestudy.net) formed the basis of recruitment of infants to BASELINE [n = 1537] and an additional 600 infants were recruited after delivery providing a total sample of 2137. The research team performed assessments on day of life 2 and at 2, 6, 12, 24 and 60 months of age. Team researchers performed specific developmental assessments at 24 months (using the Ages and Stages parental questionnaire, and the Child Behaviour Checklist) and at 60 months (using the Kaufman Brief Intelligence Test, 2nd edition (KBIT-2) and the Child Behaviour Checklist). Blood and DNA samples were bio-banked at 15 and 20 weeks' gestation, at birth, and at 24 and 60 months. Children with low scores at either time-point were examined further by the study paediatrician (DM) and were referred for early intervention assessment. In this study, archived mid-gestational (20 weeks) serum samples were analysed.

The inclusion criteria for the study were:

1. Subjects had bio-banked mid-gestational serum samples available,
2. All participants had completed 5 year follow up (ideally including developmental assessment),
3. Children who were suspected ASD cases had received a confirmed ASD diagnosis according to local practices,
4. Those participants with alternate developmental conditions (such as recognised genetic syndromes) were excluded

### Clinical diagnosis

The majority of children received their ASD diagnosis through the Health Service Executive (HSE) ASD service. The standard tests utilised in this setting are the Autism Diagnostic Observation Schedule (ADOS), and parent report via either the Diagnostic Interview for Social & Communication Disorders (DISCO) or Autism Diagnostic Interview-Revised (ADI-R) questionnaires. A small number of children received their initial diagnosis through private multidisciplinary teams using the same assessment tools. All of these children later received a confirmatory diagnosis with the HSE ASD service.

### Demographic variables

We have presented the demographic and relevant clinical data regarding the participants in Table 2. Male sex is indicated as a percentage in each participant group. Infant birthweight is presented in grams. Gestational age is in weeks. Customised birth centile indicates the percentile of the child’s birthweight in relation to their gestational age at birth. Centiles were adjusted for mothers’ height, weight at 15-week visit, ethnicity, and infant sex. The centiles were calculated using an online research calculator and were based on UK standards https://www.gestation.net/^34^. Maternal age is presented in years and sub-categorised in to three age ranges, 18–28, 29–39, > 40 years. We present maternal BMI in kg/m^2^ and sub-categorise according to WHO criteria, underweight BMI < 18.5, normal BMI 18.5–24.99, overweight BMI 25–29.99 and obese BMI > 30 kg/m^2^. We present the Apgar scores^35^ at one and five minutes as the proportion from each group with a tally less than seven. In our group there were three categories of marriage status, single, married or de facto (stable relationship akin to marriage) and finally we document smoking status in this pregnancy as (No) non-smoker, (Yes, but stopped) smoked until pregnancy was discovered, and (Still smoking) continues to smoke. The 10-question Perceived Stress Score questionnaire forms the basis for the Perceived Stress Scores (PSS). An individual’s scores on the PSS can range from zero to 40 with higher scores indicating higher perceived stress. Low stress scores range from 0–13, moderate stress scores range from 14–26, and high stress scores range from 27–40^36^. Past medical history indicates the relevant past medical history of mothers in the study, and intrapartum infections correspond to reported infections in the first 20 weeks of pregnancy.

### Ethical approval and consent to participate

Ethical approval for both the SCOPE study (Cork ECM5 (10) 05/02/08) and Cork BASELINE Birth Cohort Study (ECM3 (x) 05/04/19) were provided locally by the Cork Research Ethics Committee (CREC). We obtained written informed consent from the mothers of each case and control recruited for additional enrolment in the PiRAMiD study (Predicting early onset Autism through Maternal Immune Activation and Proteomic Discovery). Additional ethical approval for the PiRAMiD study was obtained from CREC (ECM 3 (k) 03/12/19). The Cork Research Ethic Committee (CREC) approved all research protocols in this study. Each participant gave informed consent. Clinical Research Ethics Committee of the Cork Teaching Hospitals, Lancaster Hall 6 Little Hanover Street, Cork, Ireland.

### Bio-fluid collection

We obtained archived serum samples of mothers recruited to the SCOPE-Cork study at 20 weeks’ gestation within Cork University Maternity Hospital, Cork, Ireland. Biobank specimens were archived at − 80 °C in the SCOPE (Cork) ISO accredited biobank facility until required. SCOPE study specific research midwives in accordance with best practice guidance (SCOPE Consortium S.O.P.) had performed venepuncture at the 20-week visit. Maternal specimens were collected in serum separator tubes (Becton–Dickinson Franklin Lakes, New Jersey), immediately placed on ice, and transported to the laboratory. Before proceeding to centrifugation, serum samples were stored at 4 °C for 30 min from time of collection to allow clot formation. Researchers confirmed the presence of the clot visually, and samples were then centrifuged at 2400×g for 10 min at 4 °C. Serum samples were transferred to ice cold 5 mL sterile PP (polypropylene) tubes (VWR, Radnor, Pennsylvania) via sterile Pasteur pipettes. Samples were again centrifuged at 3000×g for 10 min at 4 °C. Sera were then aliquoted to red capped, barcode-labelled cryovials (VWR) in volumes of 250 µl. Aliquots were logged in the SCOPE database (MedSciNet), and stored at − 80 °C within four hours of initial collection^37^.

### Cytokine analysis

We selected our candidate cytokines (IL-1β, IL-4, IL-6, IL-8, IL-17A, GM-CSF, TNFα, IFNγ) based on previous literature highlighting aberrations in cytokine levels in individuals with ASD^38^ versus healthy controls. We also reviewed the literature and focused on a number of publications which have measured mid-gestation (15–19 weeks) cytokines previously^30,31^, and on IL-17A in particular. Much of the recent literature espouses IL-17A’s potential as a key player in MIA associated neurodevelopmental outcomes^19,39,40^. In order to quantify IL-17A more precisely, we examined IL-17A as part of a multiplex ELISA (enzyme-linked immunosorbent assay), and then individually, using a separate ultrasensitive ELISA assay (MSD S-plex).

### MSD multiplex V-plex assay

We profiled the serologic concentrations (pg/ml) of eight cytokines and proinflammatory proteins at 20 weeks’ gestation using the Mesoscale Discovery V-plex cytokine and proinflammatory electro-chemi-luminescent (ECL) assays (Meso Scale Diagnostics, Rockville, Maryland 20850-3173, United States).

We used the V-plex multi-spot Cytokine Panel 1 (human) kit (LOT No: Z0047047) to examine IL-17A and GMCSF, and we examined IFN-γ, IL-1β, IL-4, IL-6, IL-8 and TNFα using the V-plex multi-spot Proinflammatory Panel 1 (human) kit (LOT No: Z0047096). We ran all standards in triplicate, but we ran all participant samples in duplicate due to low sample volumes.

### MSD S-plex IL-17A ultrasensitive assay

We profiled serologic concentrations (fg/ml) of IL-17A at 20 weeks’ gestation using the Mesoscale Discovery S-plex (Lot No: Z00S0003) IL-17A ECL assay (Meso Scale Diagnostics, Rockville, Maryland 20850-3173, United States). We ran all standards and participant samples in triplicate (single analyte kits require a smaller volume of serum for analysis than multiplex kits).

We performed all experiments as per the manufacturer’s instructions and analysed the plates on a MESO QuickPlex SQ 120 instrument. Numeric results were generated as “calculated concentration means” on the MSD Discovery Workbench 4.0 assay analysis software. Samples were excluded if the coefficient of variation (%CV) was higher than 25% between duplicates/triplicates as previously described^41^. We have outlined the Lower limits of detection (LLOD), lower limits of quantification (LLOQ) and the upper limits of quantification (ULOQ) as well as inter-assay CV (Coefficient of variation) for each cytokine in Table 1 for both the multiplex and ultrasensitive assays.

**Table 1:** Sensitivity of assays per each analyte examined.

	LLOD median	LLOD range	LLOQ	ULOQ	Inter-assay CV
	pg/mL	pg/mL	pg/mL	pg/mL	%
**Proinflammatory panel**					
IFNγ	0.37	0.21–0.62	1.76	938	8.16
IL-1β	0.05	0.01–0.07	0.646	375	7.95
IL-4	0.02	0.01–0.03	0.218	158	6.23
IL-6	0.06	0.05–0.09	0.633	488	8.62
IL-8	0.07	0.03–0.14	0.591	375	7.8
TNFα	0.04	0.01–0.13	0.690	248	6.74
**Cytokine panel**					
GMCSF	0.16	0.08–0.19	0.842	750	10.78
IL-17A	0.31	0.19–0.55	3.19	3650	11.45

	fg/mL	fg/mL	fg/mL	fg/mL	%
**Ultrasensitive IL-17A**					
IL-17A	13	N/A	60	140,000	8.67

In this table LLOD, LLOQ, ULOQ for each analyte tested using the MSD proinflammatory panel 1, cytokine panel 1, and MSD S-plex Human IL-17A kits. The units of measurement used in the multiplex assays are pg/ml (10^−12^ g (picograms) per millilitre), while the units in the ultrasensitive assay are fg/ml (10^−15^ g (femtograms) per millilitre). The quantitative range of the assay lies between the LLOQ and ULOQ. Inter-assay CV is a measure of the variance between runs of sample replicates on different plates and assesses plate-to-plate consistency—inter-assay CV values < 15% were deemed acceptible^42^. All inter-assay CVs were within the permissible range, indicating a low level of plate-to-plate variability.

### Statistical analysis

We compared the ASD cases (n = 25) as a whole with the neuro-typical controls (n = 50), All data were analysed using GraphPad Prism 7 (GraphPad Software Inc., San Diego, CA) and IBM SPSS Statistics 26 (SPSS Statistics, Chicago, IL). ROUT analysis^43^ was performed to remove outliers for each analyte (Q = 1%). Data arising from the cytokine analysis were analysed using Mann–Whitney U-test, as data were non-parametric. Data within the demographics table were compared using the Pearson Chi-square method for categorical data and independent t-tests or Mann–Whitney U-tests were data were parametric or non-parametric respectively. The age of the serum samples used was compared between groups using Mann–Whitney U-test. To assess for cytokine degradation over time, we correlated sample age with the concentrations of analytes using Spearman rank correlation (Rho) ρ bivariate analysis. Statistical significance (2-tailed) was set at *p* < 0.05.

## Results

**Figure 1:**
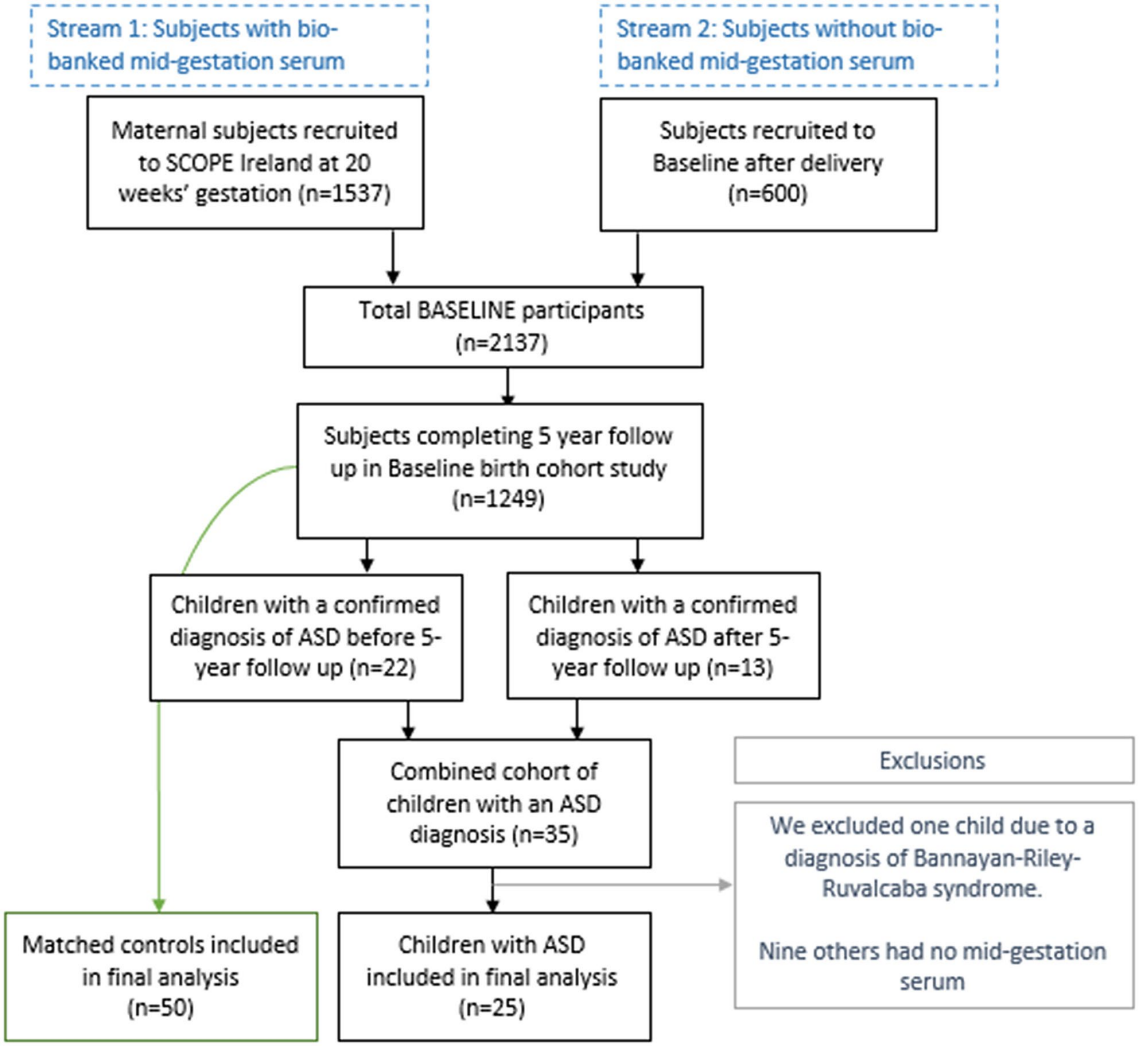
Recruitment numbers flow chart: Participants in Baseline were drawn from two streams, those recruited at 15-week booking appointment (n = 1537) and those recruited in the immediate post-natal period (600), totalling 2137 participants. 1249 participants completed follow up at 5 years. Of those, Twenty-two participants had a known ASD diagnosis at 5-year follow up; a further 13 were diagnosed with ASD between 5 and 10 years of age. Of the 35 participants with a diagnosis of ASD, 10 were excluded from this analysis. One child had a significant genetic diagnosis (Bannayan-Riley-Ruvalcaba syndrome); none of the other nine children had stored midgestational serum. This left 25 participants with ASD who were then matched 2:1 with healthy controls.

Of the initial 2137 recruited, 1249 completed 5-year follow up (see Fig. 1) Fig. 1: Included in the final analysis group were 75 child-mother pairs. Each mother had stored serum from 20 weeks’ gestation for analysis and had no significant past medical history of inflammatory disease. The case and control split was one case to two controls (25 cases to 50 controls). We selected neuro-typical, healthy controls from the same BASELINE birth cohort, and we matched controls to cases based on:

1. Infant sex,
2. Gestational age at birth,
3. Birthweight and
4. Maternal BMI at 15-week visit.

We identified 22 children with a confirmed diagnosis of ASD at the 5-year developmental assessments and a further 13 cases were diagnosed between 5 and 10 years. These “later” cases consisted of children who received their formal ASD diagnosis after 5 years of age. These cases were identified on review of the 5-year follow up documentation. Those with expressed parental concern about ASD, developmental assessment suggestive of ASD, or at risk ASD scoring in the Child Behavior Checklist (CBCL) were added to the cases cohort. The clinical research fellow verified these additional later ASD cases via a follow up telephone interview. Following confirmation of each ASD diagnosis, the research fellow invited each candidate and his or her parents to attend a follow on cognitive (KBIT-2) and ASD symptomology (SCQ) assessment. The cohort and their parents’ medical histories were further characterised using a study-specific health questionnaire.

In total, there were 10 case exclusions. Nine (9) cases had no stored serum from mid-gestation. We excluded these cases along with their matched controls. We excluded one further case (and matched controls) due to a genetic diagnosis of Bannayan-Riley-Ruvalcaba syndrome. We have depicted the recruitment stream in Fig. 1. The ASD prevalence of those 1249 children still enrolled at 5-years was approximately 3%, generally, in line with what others have quoted recently^7,44^.

### Cohort characteristics

In our cohort, the ratio of male to female ASD affected children was 4:1; this is consistent with most consensus of male preponderance in ASD^8,45^. There was no difference between groups in relation to infant birthweight or gestational age at delivery. There were no significant differences between those infants with low (< 7) reported 1- and 5-min Apgar scores. The groups matched closely in terms of maternal age and maternal BMI. All mothers participating in the study were first time mothers. The groups were ethnically homogenous, with all participants bar one of Caucasian European background. The exception was a single control of Australasian descent. With regard to inflammatory conditions and potential modifiers of inflammation, no participants reported use of any anti-inflammatories or steroids during pregnancy. Each group reported approximately equal rates of smoking. Perceived stress scores (PSS) did not significantly differ between groups, though more controls reported moderate to high stress. One mother in the control group reported suffering from Psoriasis, and a mother in the cases group reported having coeliac disease. There was no significant difference between groups in the commonly reported medical conditions of anaemia (diagnosed prior to pregnancy), asthma, depression (none on active treatment) and thyroid disease. Of those with thyroid disease, one participant from each group had hyperthyroidism; the remainder were euthyroid following treatment. In the first 20 weeks of pregnancy, 44% of controls reported at least one infection (most commonly a Respiratory Tract Infection), while only 20% of cases did so, again, this was non-significant. We have detailed the participant demographics in Table 2.

**Table 2:** Demographic characteristic of study participants.

Variable	Cases (n = 25)		Controls (n = 50)		*p*-value
	n or M	(%) or SD	n or M	(%) or SD	
Male sex	20	(80%)	40	(80%)	1
Infant birthweight	3488	SD 532	3496	SD 455	0.80
Gestational age	39.65	SD 1.5	39.78	SD 1.5	0.75
Customised birthweight centile	48.23	SD 26.5	51.90	SD 26.9	0.51
**Maternal age**	30.76	SD 5.3	31.46	SD 3.9	0.52
18–28	8	(32%)	9	(18%)	0.26
29–39	17	(68%)	39	(78%)	
> 40	0	(0%)	2	(4%)	
**Maternal BMI**	25.80	SD 4.9	25.23	SD 4.0	0.60
Underweight	1	(4%)	1	(2%)	0.25
Normal	13	(52%)	26	(52%)	
Overweight	7	(28%)	14	(28%)	
Obese	4	(16%)	9	(18%)	
Apgar 1 min < 7	4	(16%)	3	(6%)	0.16
Apgar 5 min < 7	1	(4%)	1	(2%)	0.61
**Marital status**					0.87
Single	2	(8%)	3	(6%)	
Married	20	(80%)	39	(78%)	
De facto	3	(12%)	8	(16%)	
**Smoked (pregnancy)**					0.85
No	20	(80%)	37	(74%)	
Yes, but stopped	2	(8%)	5	(10%)	
Still smoking	3	(12%)	8	(16%)	
PSS (moderate or high)	8	(32%)	24	(48%)	0.24
**Past medical history**					
Anaemia	2	(8%)	8	(16%)	0.34
Thyroid disease	4	(16%)	3	(6%)	0.26
Depression	2	(8%)	5	(10%)	0.74
Asthma	4	(16%)	5	(10%)	0.41
**Intrapartum infection (< 20w)**					
Respiratory tract infection (RTI)	3	(12%)	13	(26%)	0.16
Urinary tract infection (UTI)	2	(8%)	7	(14%)	0.45
Gastroenteritis (GE)	0	(0%)	2	(4%)	0.31

In this table, we calculated all *p*-values using the Pearson Chi square for categorical data, and independent samples t-test or Mann–Whitney U-test where appropriate for continuous variables depending on the normality of the distribution. There are no significant differences demonstrated between the groups in any of the variables listed. Cases and controls are well matched with little variance between the key matching variables, infant sex, gestational age and birthweight. Data are presented as either the mean (SD) with continuous variables or n (percentage) with categorical ones.

### Cytokine analysis

**Figure 2:**
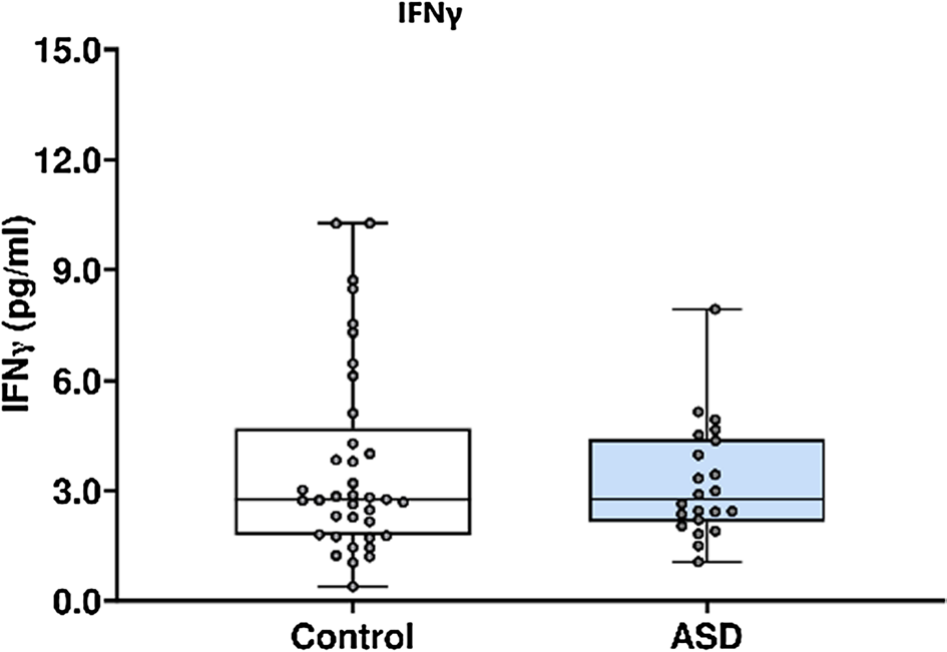
IFNγ concentration in ASD cases versus matched controls.

To determine whether a significant difference existed between the mid-gestation (20-week) inflammatory profiles of mothers of ASD and neurotypical children Meso Scale Discovery ECL assays were performed. Of the original eight cytokines examined, IL-4 was significantly altered (*p*-value 0.04). Cytokine concentrations were compared using Mann–Whitney U test. Expression of the seven other cytokines did not differ significantly. In Table 3 we present the number of samples analysed and the sample attrition rates. IL-17A was examined using a multiplex system and an ultrasensitive single analyte assay, and neither indicated a significant difference between groups. A summary of the cytokine analysis results is presented in Table 4, and the individual analyte results are presented in Fig. 2 through Fig. 10.

**Table 3:** Number of samples analysed and sample losses during processing.

Analyte	Number of samples analysed per group		Exclusions – concentration < LLOD		Exclusion – high CV	
	Cases	Control	Cases	Controls	Cases	Controls
IFNγ	24	40	0	0	1	10
IL-1β	13	15	11	31	1	4
IL-4	7	10	15	34	3	6
IL-6	25	42	0	1	0	7
IL-8	23	41	0	0	2	9
TNFα	24	42	0	1	1	7
GMCSF	8	24	13	21	4	5
IL-17A	18	36	2	6	5	8
IL-17A ultrasensitive	25	49	0	0	0	1

In this table we present the number of samples analysed for each analyte. Some of the cytokines had significant samples attrition during processing. The two reasons for loss of samples from the analysis were high CV values (> 25%) and undetectable concentrations of cytokine, below the LLOD of the assay. Use of an ultrasensitive assay rectified this issue in the case of IL-17A.

**Table 4:** Summary table of cytokine analysis results.

	Cases		Controls		*p*-value	ROUT analysis	
	Median	IQR	Median	IQR	< 0.05	Case	Control
IFN-γ	2.77	2.16–4.40	2.76	1.79–4.70	0.99	2	3
IL-1β	0.03	0.01–0.05	0.07	0.02–0.11	0.09	2	0
IL-4	0.03	0.02–0.03	0.05	0.03–0.07	0.04	1	0
IL-6	0.44	0.24–0.76	0.40	0.25–0.56	0.49	0	3
IL-8	5.52	3.90–7.00	4.88	3.52–5.79	0.10	0	5
TNFα	1.13	0.85–1.69	1.11	0.92–1.46	0.69	0	2
GM-CSF	0.12	0.08–0.28	0.16	0.10–0.24	0.38	0	2
IL-17A	0.69	0.49–0.99	0.84	0.39–1.01	0.85	0	0
IL-17A (U)	3.47	3.36–3.59	3.45	3.31–3.61	0.80	0	0

In this table we quote all analyte concentrations in pg/mL except for ultrasensitive IL-17A assay (IL-17A (U)) which we quote in fg/mL. *p*-values are statistically significant at values less than 0.05. Outliers were removed using ROUT analysis on GraphPad Prism 7 (GraphPad Software Inc., San Diego, CA). The final column “ROUT analysis” indicates the number of outliers removed from each group per analyte. We used Mann Whitney U-tests for the calculation of p-values as data were non-parametric.

### MSD multiplex V-plex

In Fig. 2 IFNγ concentration in ASD cases versus matched controls Fig. 2, IFNγ concentration was analysed using the Mesoscale Discovery platform. Units of concentration are pictograms/millilitre (pg/ml). IFNγ was not significantly altered in mothers of ASD affected children (median 2.773) at 20 weeks’ gestation compared to neuro-typical controls (median 2.763). ROUT analysis (Q = 1%) was performed to identify and exclude outliers. In total, five outliers were removed (three controls and two cases). Final analysis was performed on n = 22 cases and n = 37 controls *p* = 0.99.

**Figure 3:**
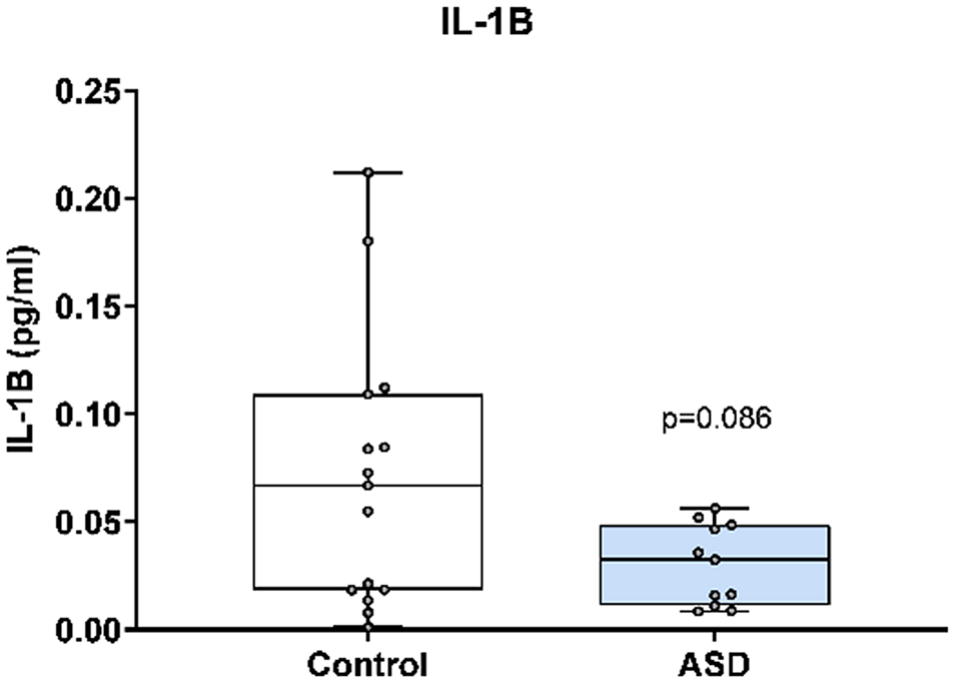
IL-1β concentration is ASD cases versus matched controls.

In Fig. 3, IL-1β concentration was analysed using the Mesoscale Discovery platform. Units of concentration are pictograms/millilitre (pg/ml). IL-1β was not significantly altered in mothers of ASD affected children (median 0.032) at 20 weeks’ gestation compared to neuro-typical controls (median 0.067). ROUT analysis (Q = 1%) was performed to identify and exclude outliers. In total, two outliers were removed (two cases). Final analysis was performed on n = 11 cases and n = 15 controls *p* = 0.09.

**Figure 4:**
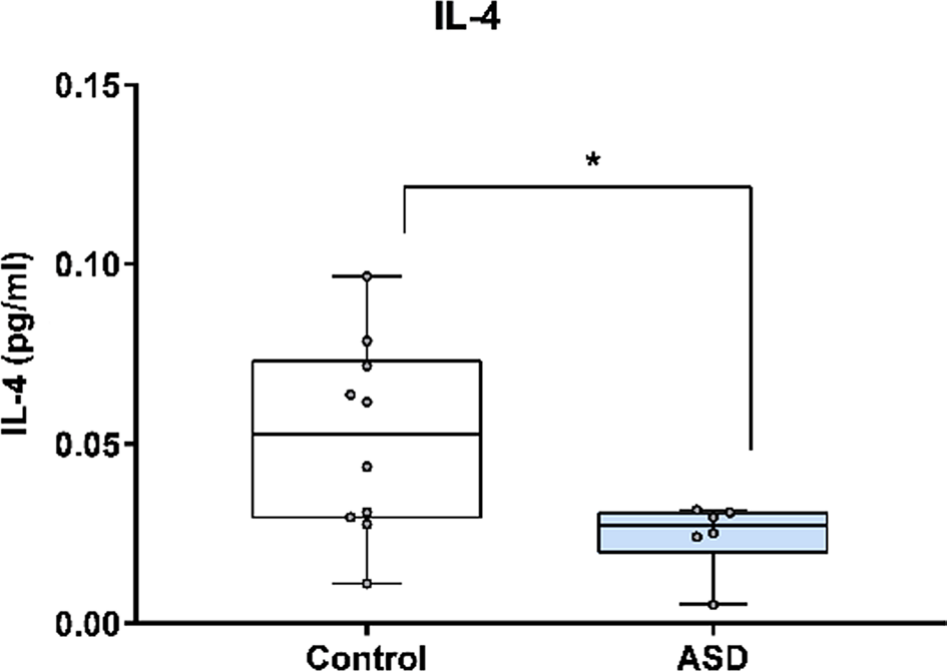
IL-4 concentration in ASD cases versus matched controls.

In Fig. 4, IL-4 concentration was analysed using the Mesoscale Discovery platform. Units of concentration are pictograms/millilitre (pg/ml). IL-4 was significantly altered in mothers of ASD affected children (median 0.027) at 20 weeks’ gestation compared to neuro-typical controls (median 0.053) *p* = 0.04. ROUT analysis (Q = 1%) was performed to identify and exclude outliers. one outlier was removed (one case). Final analysis was performed on n = 6 cases and n = 10 controls. *indicates a statistically significant *p* value < 0.05.

**Figure 5:**
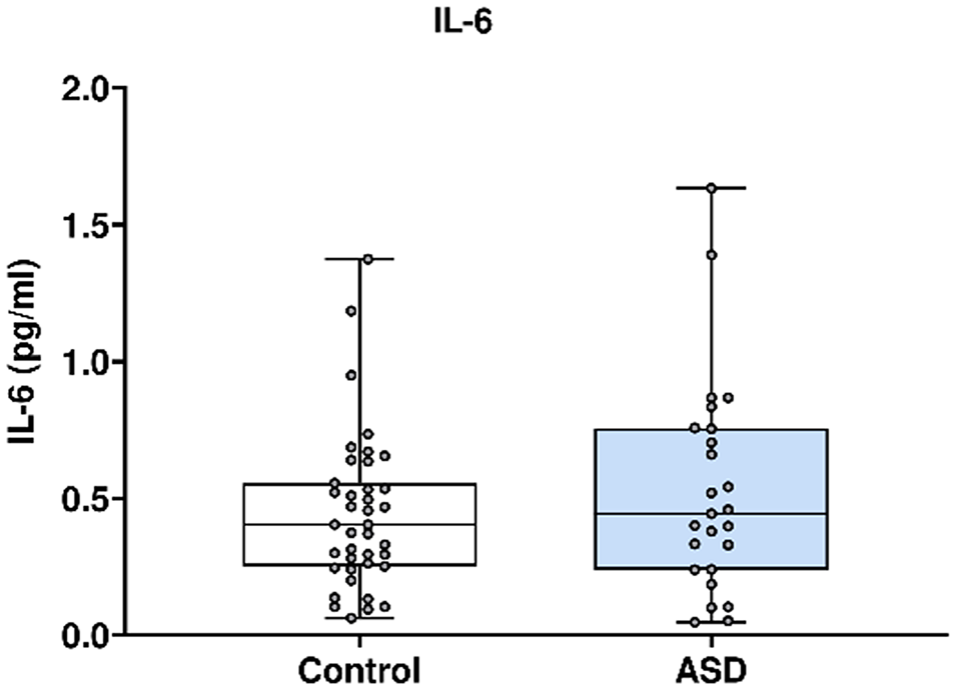
IL-6 concentrations in ASD cases versus matched controls.

In Fig. 5, IL-6 concentration was analysed using the Mesoscale Discovery platform. Units of concentration are pictograms/millilitre (pg/ml). IL-6 was not significantly altered in mothers of ASD affected children (median 0.444) at 20 weeks’ gestation compared to neuro-typical controls (median 0.404). ROUT analysis (Q = 1%) was performed to identify and exclude outliers. three outliers were removed (three controls). Final analysis was performed on n = 25 cases and n = 39 controls. *p* = 0.49.

**Figure 6:**
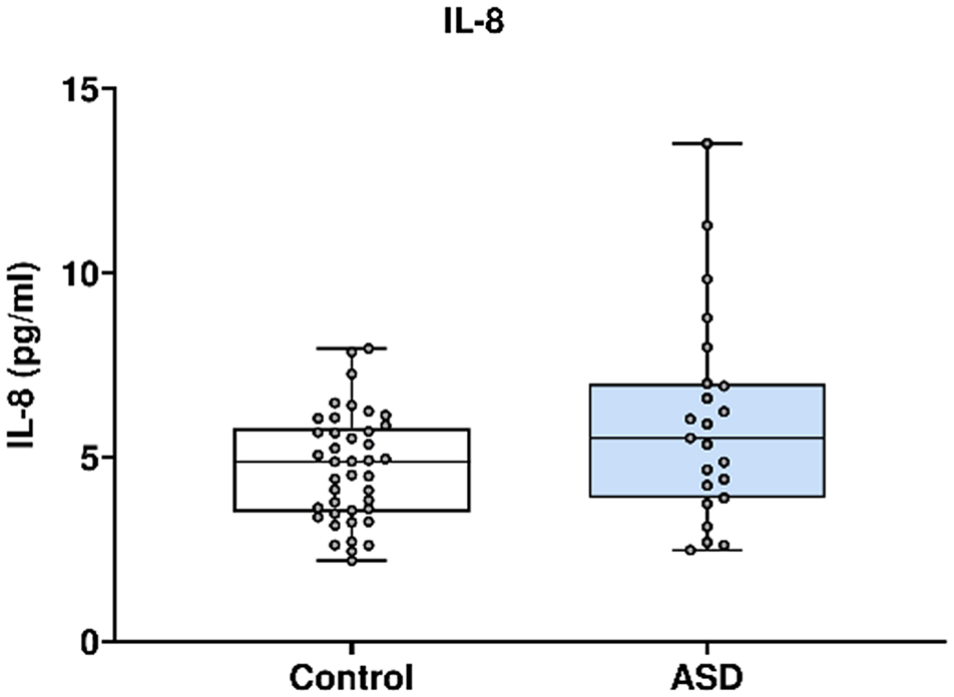
IL-8 concentration in ASD cases versus matched controls.

In Fig. 6, IL-8 concentration was analysed using the Mesoscale Discovery platform. Units of concentration are pictograms/millilitre (pg/ml). IL-8 was not significantly altered in mothers of ASD affected children (median 5.519) at 20 weeks’ gestation compared to neuro-typical controls (median 4.881). ROUT analysis (Q = 1%) was performed to identify and exclude outliers. five outliers were removed (five controls). Final analysis was performed on n = 23 cases and n = 36 controls. *p* = 0.10.

**Figure 7:**
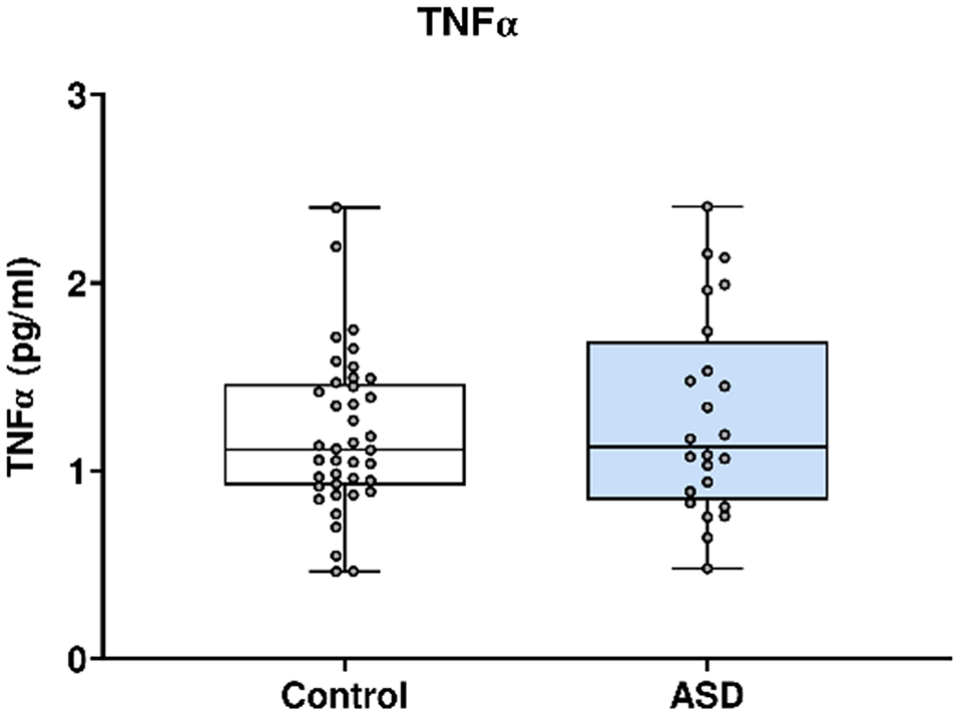
TNFα concentration in ASD cases versus matched controls.

In Fig. 7, TNFα concentration was analysed using the Mesoscale Discovery platform. Units of concentration are pictograms/millilitre (pg/ml). TNFα was not significantly altered in mothers of ASD affected children (median 1.127) at 20 weeks’ gestation compared to neuro-typical controls (median 1.114). ROUT analysis (Q = 1%) was performed to identify and exclude outliers. two outliers were removed (two controls). Final analysis was performed on n = 24 cases and n = 40 controls. *p* = 0.69.

**Figure 8:**
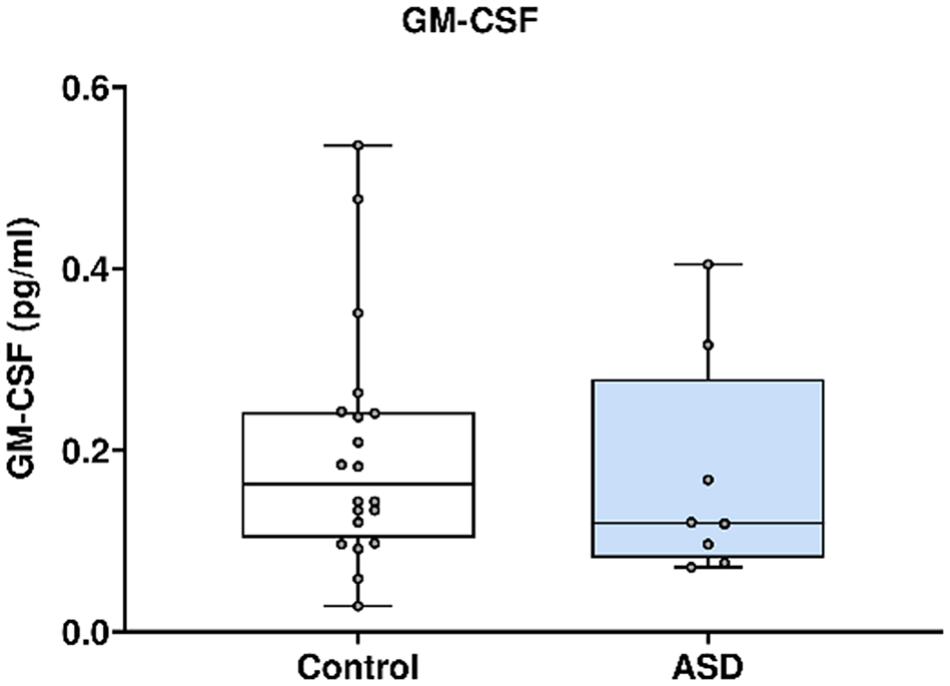
GM-CSF concentration in ASD cases versus matched controls.

In Fig. 8, GM-CSF concentration was analysed using the Mesoscale Discovery platform. Units of concentration are pictograms/millilitre (pg/ml). GM-CSF was not significantly altered in mothers of ASD affected children (median 0.120) at 20 weeks’ gestation compared to neuro-typical controls (median 0.163). ROUT analysis (Q = 1%) was performed to identify and exclude outliers. two outliers were removed (two controls). Final analysis was performed on n = 8 cases and n = 22 controls. p = 0.38.

**Figure 9:**
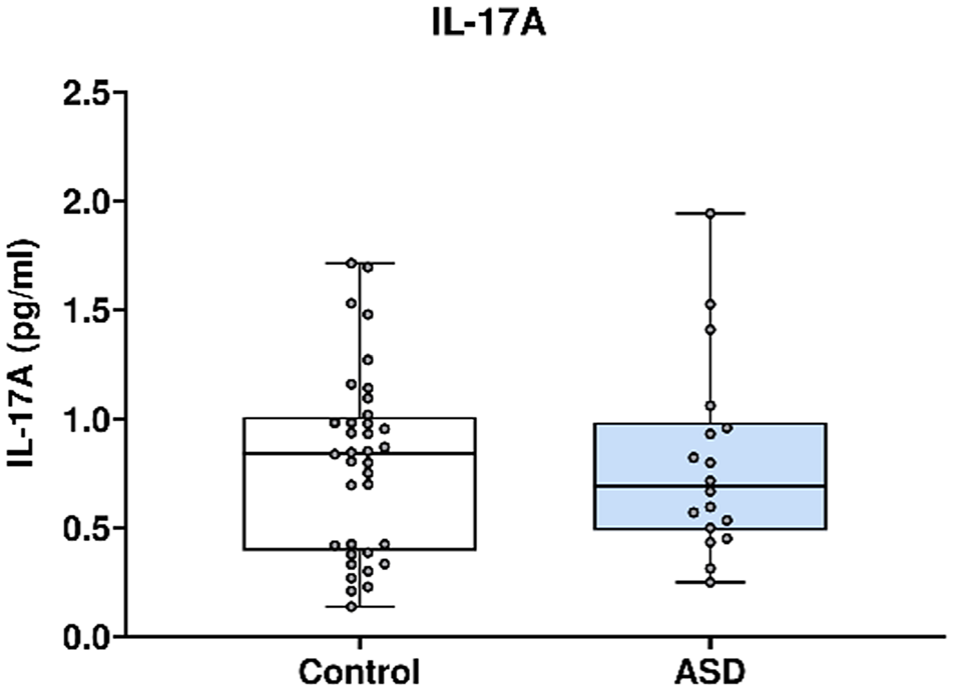
IL-17A concentrations from the multiplex analysis in ASD cases versus controls.

In Fig. 9, L-17A concentration was analysed using the Mesoscale Discovery platform. Units of concentration are pictograms/millilitre (pg/ml). IL-17A was not significantly altered in mothers of ASD affected children (median 0.691) at 20 weeks’ gestation compared to neuro-typical controls (median 0.842). ROUT analysis (Q = 1%) was performed to identify and exclude outliers, none were found. Final analysis was performed on n = 18 cases and n = 36 controls. *p* = 0.85.

### MSD S-plex ultrasensitive assay

**Figure 10:**
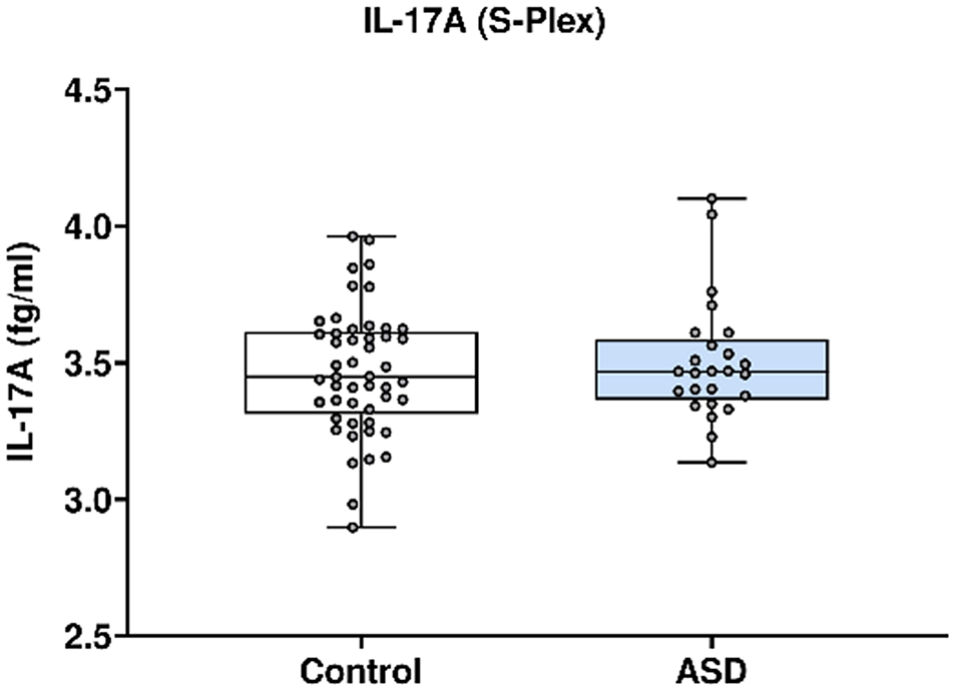
IL-17A (Ultrasensitive) concentrations of IL-17A in ASD cases versus controls.

In Fig. 10, IL-17A concentration was analysed using the Mesoscale Discovery platform. Units of concentration are femtograms/millilitre (fg/ml). IL-17A was not significantly altered in mothers of ASD affected children (median 3.468) at 20 weeks’ gestation compared to neuro-typical controls (median 3.449). ROUT analysis (Q = 1%) was performed to identify and exclude outliers, none were identified. Final analysis was performed on n = 25 cases and n = 49 controls. p = 0.80.

### Post hoc analysis to examine the effect of storage duration on sample quality

Our study used samples that had been stored for an extended time (ranging from 9.1 year to 11.8 years) before their analysis (Table 5: Sample age (years)). Somewhat mitigating this, the majority of samples were collected and stored for a similar duration before use. While degradation is highly likely to have occurred in each sample, we expect that, as all samples were stored under similar conditions, that the degree of cytokine degradation is comparable across all samples. To test this, we correlated sample age with the concentrations of each analyte (see Table 6). We found that seven of nine analytes correlated negatively with sample age suggesting some degradation over the period from the most recent to the earliest sampling. Two analytes, IL-6 and GM-CSF, correlated positively but the correlations were “weak” and “negligible” respectively. One analyte, TNFα demonstrated a significant “fair” negative correlation^46^ with sample age *ρ (Rho)* − 0.308 (p = 0.01), which is also reflected by linear regression analysis (F1, 63) = 5.037; *p* = 0.028. R^2^ = 0.074^47^. This finding confirms significant and moderate TNFα degradation in the timeframe of our sample acquisition, but no other cytokines were significantly altered in this period. While we undertook steps to reduce cytokine loss from degradation, by avoidance of freeze thaw cycles and remotely monitored ultra-low temperature storage at − 80 °C^48,49^, it remains likely that sample degradation occurred irrespective of remedial action. However, both controls and ASD samples were stored for similar lengths of time.

**Table 5:** Sample age (years).

	Total sample number	Median sample age (IQR)	*p*-value
Case	25	9.75 (9.48–10.71)	0.61
Control	50	10.08 (9.52–10.57)	

*p*-value in this table was calculated using the Mann–Whitney U test.

**Table 6:** Correlation between sample age and analyte concentration.

	IFNγ	IL-1β	IL-4	IL-6	IL-8	TNFα	IL17A	GMCSF	*IL17A
Samples (n)	64	28	17	67	64	66	54	32	74
ρ (Rho)	− 0.103	− 0.346	− 0.065	0.104	− 0.137	− 0.308	− 0.063	0.062	− 0.194
*p*	0.42	0.71	0.81	0.41	0.27	0.01	0.66	0.76	0.1

In this table, we measured correlation using Spearman’s rank correlation (Rho) ρ bivariate analysis of sample age and each individual concentration of analyte per sample. Number of samples analysed (n) per analyte. Statistical significance is considered when *p* value < 0.05. *analyte measured using ultra-sensitive MSD assay.

## Discussion

We have shown that the expression of IL-4 in maternal serum is altered significantly between ASD affected and matched control groups at 20 weeks’ gestation in a small, but carefully characterised cohort of mothers and children where the child has a diagnosis of ASD by age 10 years.

Previous evidence indicates that aberrations of the immune system may play a role in ASD^31,38,50^. Some propose that alterations in cytokine expression could facilitate the classification of ASD subtypes^24,31,51^ as well as work as biomarkers of response to treatment. In the diagnosis and management of ASD, earlier is better, and identification of reliable biomarkers during pregnancy may allow for targeted behavioural interventions from early infancy. This could also aid the development of targeted pharmacological strategies which have already shown promise in animal models^19^, and analogues of which are currently in use in routine medicine practice^52,53^.

### Interleukin-4

In demonstrating alterations in IL-4, we have corroborated findings in the small number of existing studies that have examined mid-gestational serum of mothers to autistic children. Across all of these studies (including our own), IL-4 is the only cytokine to consistently demonstrate altered expression^30,31,54^. Interestingly, while previous authors found levels of IL-4 to be elevated in the ASD affected group versus controls, in our study we found the opposite. Physiologically, IL-4 is a pleiotropic, generally anti-inflammatory cytokine that functions to suppress the pro-inflammatory milieu. Produced by activated T-cells, NK cells, and mast cell, IL-4 aids the conversion of naïve T helper cells into Th2 cells as well as potentiating the Th2 response^55,56^. IL-4 also has a role in the developmental and maintenance of key regulatory T-cells (Tregs) through STAT6 signalling pathways^57^. Tregs are important mediators of inflammation during pregnancy and at the feto-maternal interface^58^. We find IL-4 itself at the feto-maternal interface throughout pregnancy^59^, indeed in normal pregnancy; levels of IL-4 persist and increase as the pregnancy progresses^60^. Low circulating levels of IL-4 during pregnancy have been linked with spontaneous abortions, pre-eclampsia, intra-uterine growth restriction and pre-term delivery^61–63^. Failure of the usual pregnancy homeostasis (elevated IL-4 levels) may lead to a more pro-inflammatory pregnancy environment with subsequent effects on maternal health, obstetrics outcomes, and child health and development.

### Animal-based studies

Although there are very few human studies that have examined the molecular links between MIA and ASD, many animal-based studies have addressed the question of MIA and the association of elaboration of cytokines and parallel behavioural changes in offspring. MIA has been replicated in a variety of small animal models: mouse, rat and simian phenotypes of ASD have been created through intrauterine inflammatory exposure^64–66^. These models provide valuable insights into the effects inflammation can have on social and communicative behaviour in progeny^64,66^. Remedial steps have been possible with improvements in and resolution of some ASD traits following blockade of specific inflammatory pathways (IL-6 and IL-17A)^19^. This work suggested that these two cytokines in particular are significantly involved in the neuronal dysfunction brought about through MIA^19,65–67^. MIA-mouse models of ASD, have shown increased IL-17A levels in maternal blood, the postnatal brains of offspring^68^ and in placental mRNA levels of the IL-17A. This suggests upregulation of IL-17A activity at the feto-maternal interface. In 2016, Choi et al. demonstrated persuasively that simulated MIA in murine models leads to elevation in maternal IL-6, leading to downstream activation of maternal Th17 cells. Maternal Th17 cells produce IL-17A that is hypothesised to cross to the foetus via the placenta leading to increased expression of IL-17AR in the foetal brain, contributing to cortical malformations and behavioural abnormalities^19,69^.

### Human studies

Quite a number of human based studies have examined immune and cytokine aberrations in individuals (adults and children) affected by ASD themselves. Here, we will outline each of the analytes we have examined and discuss their overall function^70–72^ and highlight their purported role in ASD. TNFα is a pro-inflammatory cytokine that mediates apoptosis of virally infected cells. In previous studies, it has been demonstrated in elevated levels in the CSF and blood of ASD affected individuals^73–76^.

IL-1β is a potent pro-inflammatory cytokine involved in both acute and chronic inflammation. It has been positively correlated with ASD symptom severity^51^, as well being elevated in the serum of a number of studied ASD populations^73,74,77,78^.

IL-6 is another pro-inflammatory cytokine with broad, pleiotropic effects throughout the body (hematologic, hepatic, endocrine and metabolic)**.** It induces production of acute phase proteins and stimulates B-cell antibody production^79^. It is thought to impact synapse formation and neuronal migration^80^ as well as potentially mediating IL-17 linked ASD risk in pregnancy^19,66^. Alterations in expression have been noted in the serum and CSF of autistic individuals^73,74,76–78,81,82^.

IFNγ is a versatile cytokine that interfaces between innate and adaptive immune response. It is secreted by NK cells, and promotes NK killing. It activates macrophages, which in turn, produce IL-12 and -23, stimulating Th1 and Th17 cell respectively. IFNγ inhibits Th2 cells and plays a role in defence against intracellular pathogens, tumour surveillance, autoimmunity, allergy and the protection of the amniotic space during pregnancy^83^. A number of studies have identified alteration of IFNγ in ASD groups^38,73,81^.

IL-17 is a pro-inflammatory and chemotactic cytokine. Derived from Th17 cells, a subset of CD4 cells, IL-17 potentiates the innate polymorphonuclear cell response throughout inflammation. In ASD studies, it is postulated to trigger alterations in the blood brain barrier and lead to cortical dysplasia^66^, and altered concentrations of IL-17 have been identified in the sera of ASD affected individuals^24,26,73,76,82,84^.

GM-CSF is a colony stimulating growth factor that is produced by stromal cell. It targets bone marrow, and precursor cells, mediating haematopoiesis. In one study, altered levels have been observed in individuals with ASD^85^. IL-8 is classified as a chemotactic cytokine and is produced by fibroblasts, neutrophils and macrophages. It is chemo-attractant for phagocytes at site of inflammation, and has been identified in a number of studies as altered in ASD populations versus controls^27,77,81^. While the cytokine profiles of ASD affected individuals have been well characterised, very few studies have investigated the relationship between mid-gestation cytokine levels and ASD risk in offspring. To our knowledge, only three human studies have examined maternal serum^30,31,54^, and one more has examined amniotic fluid cytokine profiles in mothers of ASD affected children^86^. The findings from these studies, effectively provide all of our current understanding of gestational cytokine profiles in the setting of ASD.

### Previous literature on gestational samples analysis in ASD

Working from the same laboratory and using similar methods, Goines et al. (2011) and more recently, Jones et al. (2017) both demonstrated elevated mid-gestational cytokine levels between groups of ASD affected children versus controls or children without ASD. Goines et al. demonstrated elevated levels of mid-gestation (15–19 weeks’ gestation) IFNγ, IL-4 and IL-5 with an associated 50% increased ASD risk. While Jones et al. showed elevated levels of mid-gestation GM-CSF, IL-6, IFNγ and IL-1α in the ASD affected group versus children with developmental delay, but not ASD. The authors do not mention the age of the samples used in either study, but the samples used were sourced from the same birth cohort in Orange County, California between 2000 and 2003. In both studies, the samples were initially stored at room temperature and later at − 20 °C freezer conditions before long-term storage at − 80 °C. This initial handling may have contributed to some cytokine degradation. In the Goines study, ASD cases were matched with neuro-typical controls based solely on child characteristics (sex, birth month and year), something which the authors acknowledge in their limitations. Neither study had access to comprehensive maternal health information during the pregnancy (including intrapartum infections). Nor did they have a record of relevant maternal medical history, all, information important to the interpretation of their findings.

Irwin et al. (2018) demonstrated alterations in IL-4, MCP-1 and IL-10 levels in 28-week gestation serum of mothers who birthed ASD affected children^54^. Specifically, IL-4 (usually anti-inflammatory or involved in allergic type inflammation^87^) was increased and associated with higher ASD symptomology (as measured by the Social Communication Questionnaire (SCQ)) in offspring. Higher concentrations of IL-10 (anti-inflammatory) were associated with fewer ASD symptoms in offspring (measured by the Social Responsiveness Scale (SRS)), and finally, elevated MCP-1 was associated with fewer ASD symptoms (as measured by the SCQ). The samples used in this analysis were reported to be at least 5 years old. No controls were used in this analysis, instead a large cohort of ASD affected individuals were enrolled, and the 28-week gestation cytokine concentrations were correlated with ASD symptomology at 7 years of age. This is novel in two senses, none has previously assessed the cytokine profile in the third trimester, and none has correlated cytokine findings with severity of ASD symptomology in this way. As with previous authors, they had no access to relevant maternal pre-conceptual medical history or gestational infections data.

Finally, Abdallah et al. (2013) examined amniotic fluid samples and found elevated levels of IL-4, IL-10, TNFα, and TNFβ. In a preliminary study (2012), they also identified elevations in MMP-9 in ASD cases relative to controls^88^. Advanced sample age is again an issue with the oldest samples in this analysis being 29 years old, the youngest 10 years old. The samples were stored at − 20 °C according to local guidance^89^. Both the storage conditions and the samples ages are likely to have contributed to significant cytokine degradation^48^.

### Limitations

The samples used in our study fall outside the ideal sample age for accurate analysis of cytokines^48^. To our mind, this is the single most important limitation confronting studies of this nature. Unfortunately, the shelf life of archived samples is finite, and even samples in long-term ultra-low temperature storage (− 80 °C) suffer from degradation of cytokines and chemokines over time^48,49^. Retrospective sample analysis, would present an excellent opportunity to study cytokine aberrations in ASD, if the time to ASD diagnosis was shorter. One UK study found that the average delay between concerns first being noted by parents and the child receiving a diagnosis of an ASD was 4.6 years (SD 4.4 years)^90^. ASD services continue to be under-resourced^91^ and diagnoses are chronically delayed^92^. Under current conditions, our experience of retrospective analysis of archival samples suggests that this style of study design is not well suited to addressing this question. Even large-scale population based studies would suffer from the same issues of sample fidelity over longer periods.

To ensure future study designs are capable of accurate mid-gestation cytokine analysis, they should be prospective, and concentrate on early ASD case identification or screening. Early identification should be paramount, the diagnostic stability of ASD is reliably fixed from as early as 14 months old^93^ so screening and identification within the first 2–3 years of life is possible. Cytokines should be analysed contemporaneously, acute phase reactants such as IL-1β and IL-6 have demonstrated greater than 50% degradation within 3 years even in − 80 °C freezer conditions^48^. IL-4 is stable only for 3 years, while IL-17A, IFNγ, and TNFα, all suffer more than 50% degradation within 4 years at ultra-low temperature storage^48^. Basic handling of samples and initial processing requires optimisation to ensure the risk of sample degradation is minimised: (i) Store samples at ultra-low temperatures, (ii) initial processing should be rapid (< 1 h from venepuncture to freezer storage) and (iii) freeze-thaws cycles should be minimised. With robust methods of early screening in place, early confirmatory diagnosis within the first 2/3 years, and analysis of gestational samples within 3 years, it should be feasible to increase the yield and validity of such studies, and greatly reduce cytokine loss through prolonged storage. While this approach would allow for study of children presenting with the earliest signs of ASD, or targeted high-risk groups (ASD affected siblings). It would likely miss those presenting later, including those who are a high-functioning phenotype or of female sex.

Finally, our small sample size is a major limitation, and results should be interpreted with caution. Analysis of IL-4 levels in the groups yielded results on only 16 individuals (6 cases and 10 controls). Attrition of the viable samples was due to a combination of the low absolute concentrations of IL-4 in the samples (likely exacerbated by advanced sample age), concentrations at or below the sensitivity (LLOD) of the MSD multiplex format and high CV values. It is difficult to make inferences about results in samples this size, and larger scale group analysis is warranted.

### Strengths

Although our study has suffered from some of the same limitations as previous studies, our study is strengthened by the quality of our cohort. Each child had a concrete specialist service ASD diagnosis, confirmed by the clinical paediatric fellow. Each child was well characterised clinically and matching was strictly observed. Matching was not only based on child characteristics (Sex, Gestational age, Birthweight), but also on an important maternal characteristic, BMI at 15 weeks’ gestation. This enhanced the validity of our results. In addition to detailed child characteristics, we have also included important information regarding the past medical histories, medication or anti-inflammatory use, and pre-existing inflammatory conditions of the mothers included in the study. We present crucial information about infection rates in the first 20 weeks of pregnancy, all of which presents a major confounder to accurate analysis if this information is absent. Our methods were robust, and we identified two key issues of multiplex assay sensitivity and advanced sample age, and remedied the former through utilisation of ultrasensitive single analyte plates.

## Conclusion

In conclusion, in a carefully characterised maternal-child cohort study we did not replicate the findings of similar mid-gestational studies, but did find some evidence of mid-gestational cytokine aberrations (downregulated IL-4) in the mothers of children with ASD. Reduced levels of IL-4 are linked to a pro-inflammatory state during pregnancy and negative obstetric and foetal outcomes. All studies to date have had similar and significant limitations. Future studies should focus on minimising the time between sample acquisition and analysis, use of best practice for initial sample handling, and early identification and characterisation of cases and their mothers. Future analysis should be serial and include investigation of samples taken from early in pregnancy. The first trimester, and particularly 8–12 weeks’ gestation is a crucial period for organogenesis and differentiation, and analysis from this period will help complete the picture of gestational cytokine fluctuations and their effect on neurodevelopment.

## Data Availability

The datasets used and analysed during the current study are available from the corresponding author on reasonable request.

## Abbreviations

ADI-R: Autism diagnostic interview-revised
ADOS: Autism diagnostic observation schedule
ASD: Autism spectrum disorder
BMI: Body mass index
CBCL: Childhood behavioral checklist
CD 4 cell: Cluster of differentiation 4 cell
CREC: Cork research ethics committee
CSF: Cerebrospinal fluid
DISCO: Diagnostic interview for social and communication disorders
ECL: Electrochemiluminescence
ELISA: Enzyme-linked immunosorbent assay
GE: Gastroenteritis
GMCSF: Granulocyte–macrophage colony-stimulating factor
HLA-G gene: Human leukocyte antigen G coding gene
HSE: Health service executive
ID: Intellectual disability
IL-1α, IL-1β, IL-2, IL-4, IL-6, IL-8, IL-10 and IL-17A: Interleukins 1 alpha, 1 beta, 2, 4, 6, 8, 10, 17A
IL-17AR: Interleukin 17A receptor
IQR: Interquartile range
ISO: International Organization for Standardization
KBIT-2: Kaufmann brief intelligence test, second edition
LLOD: Lower limit of detection
LLOQ: Lower limit of quantification
MIA: Maternal immune activation
mRNA: Messenger ribonucleic acid
MSD: MesoScale discovery
MZ: Monozygotic
Poly (I:C): Polyinosinic: Polycytidylic acid
PMN: Polymorphonuclear cells
PP: Polypropylene
PSS: Perceived stress score
ROUT: Robust regression and outlier removal
RTI: Respiratory tract infection
SCQ: Social communication questionnaire
SOP: Standard operating procedure
SRS: Social responsiveness scale
STAT6: Signal transducer and activator of transcription 6
Th1, 2 and 17: T helper 1, T helper 2, and T helper 17 cells
TNFα, TNFβ: Tumour necrosis factor alpha, beta
Tregs: Regulatory T-cells
ULOQ: Upper limit of quantification
UTI: Urinary tract infection
